# Widespread, depth-dependent cortical microstructure alterations in pediatric focal epilepsy

**DOI:** 10.1111/epi.17861

**Published:** 2023-12-26

**Authors:** Chiara Casella, Katy Vecchiato, Daniel Cromb, Yourong Guo, Anderson M. Winkler, Emer Hughes, Louise Dillon, Elaine Green, Kathleen Colford, Alexia Egloff, Ata Siddiqui, Anthony Price, Lucilio Cordero Grande, Tobias C. Wood, Shaihan Malik, Rui Pedro A.G. Teixeira, David W. Carmichael, Jonathan O’Muircheartaigh

**Affiliations:** 1 Centre for the Developing Brain, School of Biomedical Engineering and Imaging Sciences, King’s College London, London, UK; 2 Department for Forensic and Neurodevelopmental Sciences, Institute of Psychiatry, Psychology, and Neuroscience, King’s College London, London, UK; 3 Department of Human Genetics, University of Texas Rio Grande Valley, Brownsville, Texas, USA; 4 Department of Radiology, Guy’s and Saint Thomas’ Hospitals NHS Trust, London, UK; 5 Department of Biomedical Engineering, King’s College London, London, UK; 6 Biomedical Image Technologies, Telecommunication Engineering School (ETSIT), Technical University of Madrid, Bioengineering, Biomaterials and Nanomedicine Networking Biomedical Research Centre, National Institute of Health Carlos III, Madrid, Spain; 7 Department of Neuroimaging, King’s College London, London, UK; 8 Medical Research Council (MRC) Centre for Neurodevelopmental Disorders, London, UK

**Keywords:** cortical microstructure, epilepsy, focal cortical dysplasia, MRI, relaxometry

## Abstract

**Objective:**

Tissue abnormalities in focal epilepsy may extend beyond the presumed focus. The underlying pathophysiology of these broader changes is unclear, and it is not known whether they result from ongoing disease processes or treatment-related side effects, or whether they emerge earlier. Few studies have focused on the period of onset for most focal epilepsies, childhood. Fewer still have utilized quantitative magnetic resonance imaging (MRI), which may provide a more sensitive and interpretable measure of tissue microstructural change. Here, we aimed to determine common spatial modes of changes in cortical architecture in children with heterogeneous drug-resistant focal epilepsy and, secondarily, whether changes were related to disease severity.

**Methods:**

To assess cortical microstructure, quantitative T1 and T2 relaxometry (qT1 and qT2) was measured in 43 children with drug-resistant focal epilepsy (age range = 4–18 years) and 46 typically developing children (age range = 2–18 years). We assessed depth-dependent qT1 and qT2 values across the neocortex, as well as their gradient of change across cortical depths. We also determined whether global changes seen in group analyses were driven by focal pathologies in individual patients. Finally, as a proof-of-concept, we trained a classifier using qT1 and qT2 gradient maps from patients with radiologically defined abnormalities (MRI positive) and healthy controls, and tested whether this could classify patients without reported radiological abnormalities (MRI negative).

**Results:**

We uncovered depth-dependent qT1 and qT2 increases in widespread cortical areas in patients, likely representing microstructural alterations in myelin or gliosis. Changes did not correlate with disease severity measures, suggesting they may represent antecedent neurobiological alterations. Using a classifier trained with MRI-positive patients and controls, sensitivity was 71.4% at 89.4% specificity on held-out MRI-negative patients.

**Significance:**

These findings suggest the presence of a potential imaging endo-phenotype of focal epilepsy, detectable irrespective of radiologically identified abnormalities.

## 1 Introduction

Increasing evidence suggests that common epilepsies share disturbances in distributed corticosubcortical brain networks.^1–8^ However, the pattern, consistency, and cause of these disturbances are currently unknown, as it is uncertain how they may relate to clinical parameters such as functional decline.^9–11^

In focal epilepsy, subtle but widespread structural abnormalities have been demonstrated in cortical areas remote from the putative epileptic focus.^12–15^ Such cortical changes may be attributed to the adverse effects of medication,^16,17^ and ongoing and cumulative disease processes.^18,19^ Alternatively, it is possible that such alterations represent a predisposing factor to disease development.^13,20^

Most previous magnetic resonance imaging (MRI) studies in epilepsy have assessed structural morphological changes, including volume and cortical thickness.^8,21^ However, morphological markers reflect the combination of several neuroanatomical features.^22^ Quantitative MRI (qMRI) techniques such as longitudinal (T1) and transverse (T2) relaxometry (qT1 and qT2) are sensitive to tissue structure and biophysical properties at the micrometer scale,^23^ and allow the in vivo assessment of inconspicuous changes in cortical tissue properties.^24^ In particular, qT1 and qT2 reflect tissue density, macromolecule, protein, and lipid composition, and iron concentration. Importantly, because they are quantitative, these metrics can be compared between studies and in longitudinal investigations.^25^

In adults, previous research revealed qT1 increases in ipsilateral temporal and frontal limbic cortices in temporal lobe epilepsy (TLE), with more marked effects in upper cortical levels that taper off toward the white/gray matter (WM/GM) boundary. Increases were particularly marked in patients with early disease onset, suggesting that these alterations may reflect atypical neurodevelopment.^26^

Ahmad et al.^21^ showed widespread cortical qT2 increases in normal-appearing cortical tissue in frontal, parietal, and temporal regions of patients with focal cortical dysplasias (FCDs), demonstrating effects of the disease in cortical regions beyond the identified focus.

Previous research also revealed the usefulness of qT1 and qT2 for the detection of FCDs in adult patients, for example, by assessing the cortical extent and the smoothness of qT1 at the WM/GM junction^27^ or the SD of qT1 and qT2 values.^28^

Other studies moved beyond the neocortex and applied qT2 to study hippocampal changes in adults with TLE. For example, Bernasconi et al. demonstrated that hippocampal qT2 changes are present in adults with TLE even when no atrophy is detected and can lateralize the epileptic focus in most patients.^29^ Winston et al.^30^ showed that the combination of hippocampal volumes and qT2 values can reliably identify sclerotic hippocampi in TLE.

There is relatively little neuroimaging research in pediatric epilepsy. Importantly, the application of qMRI may enable improved detection of the microstructural processes related to tissue remodeling in neurodevelopment, thus allowing better separation of microstructural processes related to neurodevelopmental changes from the effects of the disease.^25,31^

A recent study^4^ evaluated the distribution of whole-brain qT1 and qT2 in a sample of both children and adults with pharmacoresistant focal epilepsy and no apparent lesions on conventional MRI. The authors reported multiregional qT1 increases in patients, in both GM and WM ipsilateral to the epileptic origin, involving limbic/paralimbic systems. qT1 increases correlated with younger seizure onset age, longer epilepsy duration, and higher seizure frequency, suggesting that such changes may be related to seizure burden.

In this study, we investigated surface-based qT1 and qT2 values at increasing cortical depths,^32^ as well as their gradient of change as an index of microstructural organization^26^ in a heterogeneous sample of children with drug-resistant focal epilepsy. Assessing a wide range of patients with diverse underlying pathologies allowed deeper insight into whether biologically distinct focal epilepsy syndromes show robust, common microstructural deficits. As children typically have shorter disease duration and therefore less opportunity for chronic disease-related cortical damage, we expected to detect focal, rather than global, cortical abnormalities.^26^ The relationship of these relaxation parameters to age at onset, disease duration, and number of seizures per year was also tested.

Furthermore, we explored the potential of qMRI features to act as a biomarker of focal epilepsy, even when there is no radiologically identified focus. For this purpose, we trained a classifier using whole-brain qT1 and qT2 cortical features from MRI-positive patients and healthy controls, and tested whether the resulting classifier could identify MRI-negative patients and controls held out from the training phase.

## 2 Materials and Methods

### 2.1 Subjects

Participants were recruited prospectively, and ethical approval was granted by the Health Research Authority and Health and Care Research Wales (ethics ref. 18/LO/1766). Infants younger than 6 months and young adults older than 18 years were excluded, as were people with major neurological conditions unrelated to epilepsy and contraindications for 3-T MRI.

Eligible patients were recruited through the pediatric neurology outpatient clinics at Evelina London Children’s Hospital, King’s College Hospital, and Great Ormond Street Hospital, whereas children without epilepsy were recruited from existing volunteer databases, schools, and the King’s College London recruiting webpage.

For participants younger than 16 years, written signed consent was obtained from the parents (or the person with legal parental responsibility) prior to data collection. Adolescents 16 years or older provided their own written consent.

A total of 96 children were recruited for the present study, 89 of whom were included in the analysis: 43 with drug-resistant focal epilepsy (age range = 4–18, mean = 12 years, 22 female) and 46 healthy controls (age range = 2–18, mean = 11.5 years, 27 female). Of the seven participants excluded, four presented incidental findings and three had missing data. There was no significant difference in age between patients and controls (*p* =.68).

In patients, diagnosis and lateralization of the seizure focus were determined by a comprehensive evaluation including detailed history, neurological examination, review of medical records, video-electroencephalographic recordings, and clinical MRI evaluation. Among the patients assessed, the majority had temporal involvement, followed by frontal, occipital, parietal, and occipital and parietal involvement (Table 1). A detailed description of the patient sample is provided in Table S1.

### 2.2 Image acquisition

Children were scanned without sedation on a 3-T Achieva-TX (Philips Healthcare) using a 32-channel head coil. The protocol included a three-dimensional (3D) T1-weighted magnetization-prepared rapid acquisition gradient echo (MPRAGE) acquisition (repetition time [TR] = 7.7 ms, echo time [TE] = 3.6 ms, flip angle = 8°, inversion time [TI] = 900 ms, echo-train length = 154, acquisition time = 286 s); a T2-weighted fluid-attenuated inversion recovery (FLAIR) acquisition (TR = 5000 ms, TE = 422 ms, TI = 1800 ms, echo-train length = 182, acquisition time = 510 s); and a joint system relaxometry (JSR) acquisition,^33^ consisting of two spoiled gradient recalled sequences (flip angle = 2.2° and 12.5°, TR = 6.2 ms) and three balanced steady-state free precession (bSSFP) sequences (with a 12° flip angle and a 180° phase increment, 49° flip angle and 0° phase increment, and 49° flip angle and 180° phase increment, respectively; all had a TR of 6 ms). Parallel imaging acceleration (SENSitivity Encoding, SENSE) of 1.4 was used along both phase-encoding directions. The field of view was 240 × 188 × 240 mm, and the resolution was 1 mm isotropic for all images.

### 2.3 Motion tolerance

All scans were acquired using the DISORDER scheme, which has demonstrated improved tolerance against motion (Figure S1).^34,35^ In-depth detail of the reconstruction algorithm has been described previously.^34,36^

### 2.4 Image analysis

**Figure 1:**
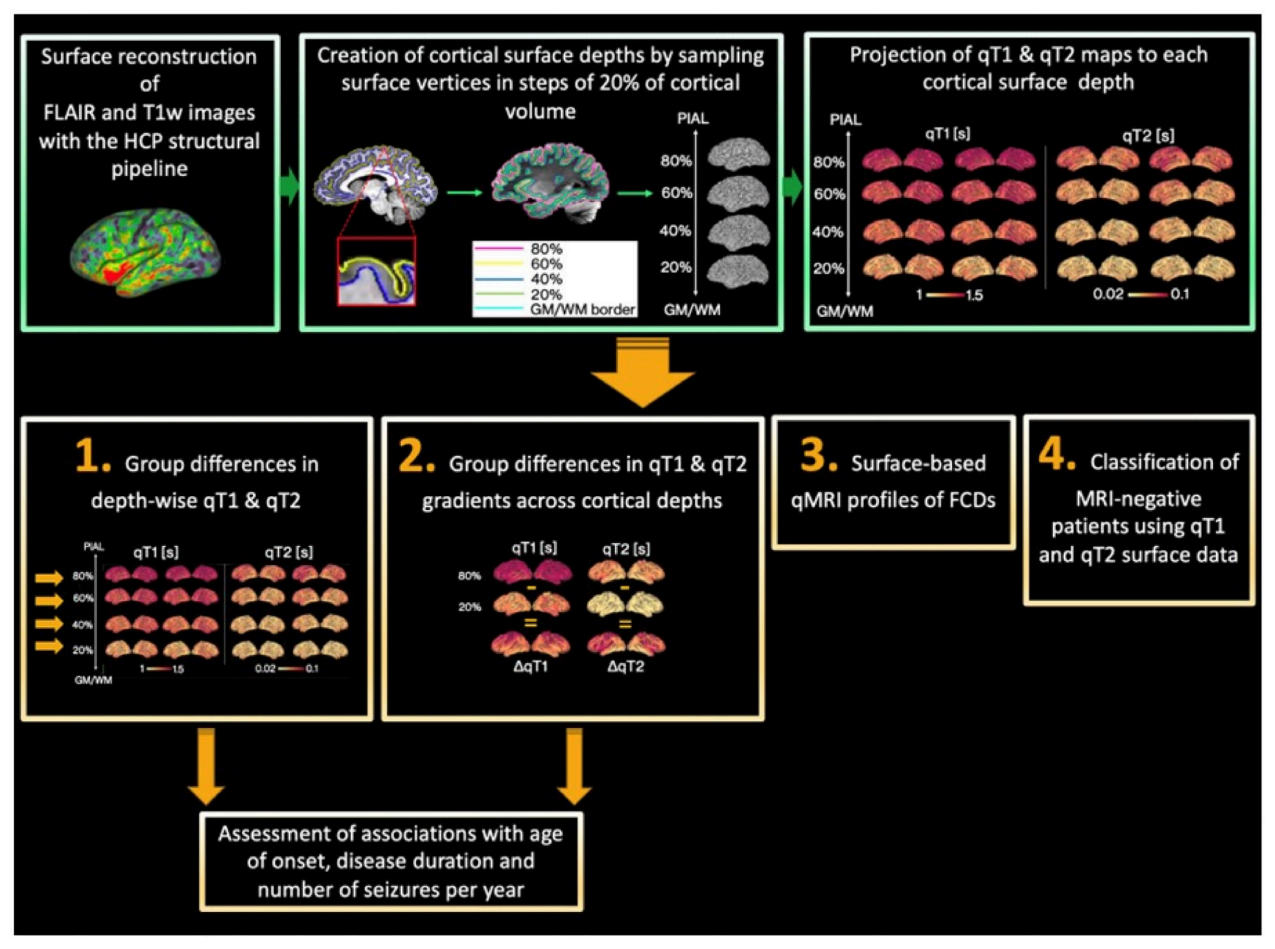
Diagram of data processing (green boxes) and analysis (orange boxes). First, surface reconstruction was performed with the Human Connectome Project (HCP) structural pipeline.^32^ Then, surface extraction was performed at a series of cortical depths, and qT1 and qT2 maps were projected onto each depth. This allowed the assessment of group differences in (1) qT1 and qT2 values at several depths from the pial surface and (2) qT1 and qT2 gradients across cortical depths. Associations with age at onset, disease duration, and number of seizures per year were also explored. Additionally, we computed surface-based quantitative magnetic resonance imaging (qMRI) profiles of focal cortical dysplasia (FCD) lesions (3) and assessed whether cortical microstructure patterns learned from MRI-positive patients could classify MRI-negative patients (4). FLAIR, fluid-attenuated inversion recovery; GM/WM, gray/white matter; T1w, T1-weighted.

Image analysis steps are summarised in Figure 1.

#### 2.4.1 Surface reconstruction

FLAIR and MPRAGE images were coregistered and jointly analyzed with the Human Connectome Project (HCP) structural pipeline^32^ to reconstruct WM/GM boundaries and pial surfaces. Preprocessing steps and the code to run the HCP pipeline can be found at https://github.com/Washington-University/HCPpipelines.

These surfaces were used to compute equivolume cortical surface depths by sampling vertices in steps of 20% of cortical volume (0%: WM/GM boundary, 100%: pial surface) across the entire cortex in Connectome Workbench (https://github.com/Washington-University/workbench).

#### 2.4.2 qT1 and qT2 surface mapping

JSR^33^ fits were performed within the QUIT toolbox,^37^ providing 3D qT1 and qT2 maps. qT1 and qT2 images were rigidly coregistered to the FLAIR and MPRAGE volume, smoothed (sigma =.4), and sampled at each cortical depth using the ribbon mapping method in Connectome Workbench. This constructs a polyhedron from the vertex’s neighbors on each surface and estimates the amount of this polyhedron’s volume that falls inside nearby voxels, to use as weight for sampling.

#### 2.4.3 Group differences in depthwise cortical qT1 and qT2 and in their gradient of change across cortical depths

The HCP structural pipeline outputs cortical surface metrics that are left–right symmetrical, meaning that a vertex with a specific number will refer to roughly homologous areas in each hemisphere.^32^ Therefore, we flipped the qT1 and qT2 surface maps of patients with right hemispheric focus so that they could be analyzed with patients with left hemispheric focus. This enabled both maximizing sample size and better understanding the impact of focus laterality on detected changes.

Groupwise differences at 20%, 40%, 60%, and 80% cortical distance from the GM/WM border to the pial surface were tested. Additionally, at each vertex, qT1 and qT2 values at 20% distance were subtracted from those at 80% distance, providing a gradient of relaxation values across the cortex, here an index of intracortical organization. Group differences in this gradient were tested. Finally, associations between qT1 and qT2 changes in patients with age at disease onset, disease duration, and number of seizures per year were tested. Because of skewness in the data, the number of seizures per year data was log-transformed before assessing correlations. All models were tested using the Permutation Analysis of Linear Models (PALM) package.^38^

To understand whether the focal cortical location had a significant impact on any observed changes, the above analyses were repeated by splitting patients with temporal focus (*n* = 22) and patients with frontal focus (*n* = 13) into two subgroups. Here, we excluded patients with occipital (*n* = 3) and parietal (*n* = 4) foci, because of low numbers, as well as those patients who presented with both occipital and temporal foci (*n* = 1).

As a first step, for all analyses, we tested for an interaction between age and group. Where this was not significant, age and sex were regarded as covariates of no interest in the final analyses. Vertexwise cortical thickness was also controlled for in the analyses, because of evidence that this feature is altered in epilepsy.^12,15^ Finally, as differences in cortical curvature have been shown to systematically affect the laminar and myeloarchitecture of the cortex,^40^ we also controlled for vertexwise cortical curvature. To control for multiple comparisons, familywise error (FWE) correction rate was applied in each analysis separately across modalities (i.e., qT1 and qT2) and statistical contrasts (i.e. patients > controls and controls > patients) using the “-corrmod” and “-corrcon” options in PALM. Threshold-free cluster enhancement was employed as test statistic,^39^ and relationships were tested by permutation using the Draper–Stoneman method,^38^ which ensures that spatial dependencies in the data are preserved for all permutations.

#### 2.4.4 Surface-based qMRI profiles of individual FCDs

**Figure 2:**
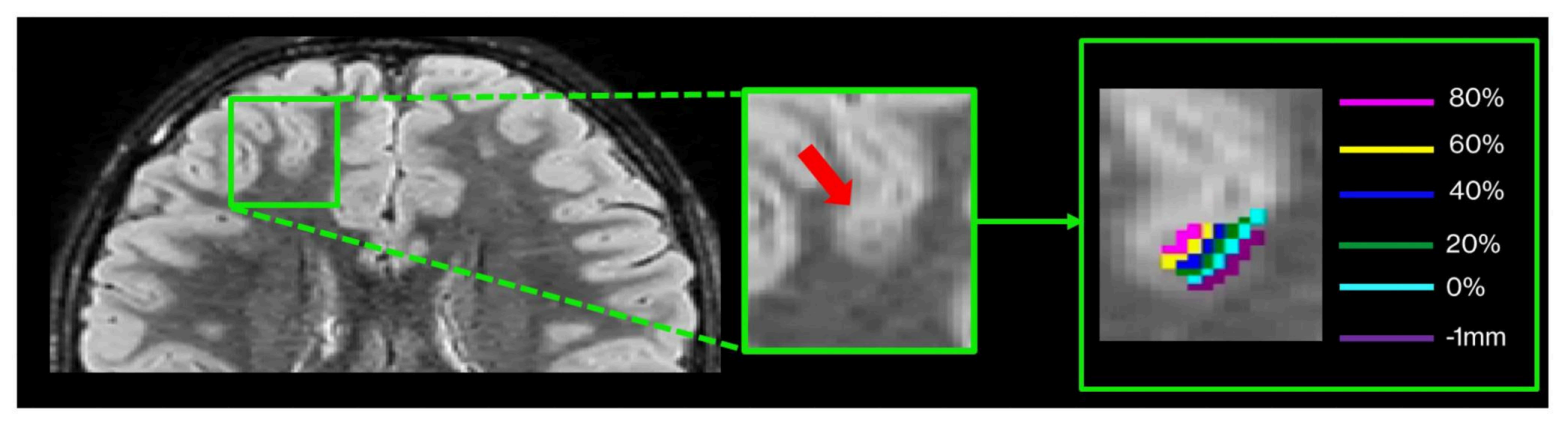
Surface-based quantitative magnetic resonance imaging profiles of focal cortical dysplasias (FCDs). Lesions were delineated on the fluid-attenuated inversion recovery images of each of the five patients with FCDs. The lesion masks were then mapped to each cortical depth in patients and controls in standard surface space. The mean and SD of qT1 and qT2 were extracted within the masks, after removing the effect of age and sex using linear regression. This allowed computation of the *z*-score profile of each patient normed against the controls, corrected for age and sex.

To assess whether changes seen in group differences may have been driven by focal lesions in individual patients, we performed a post hoc examination of the qMRI profiles of lesions of five patients with radiologically suspected FCDs. For this purpose, we first manually delineated lesion masks on the FLAIR images in each patient. We then mapped the lesion masks to each cortical depth in both patients and controls in the HCP standard surface space, consisting of approximately 32 000 vertices (Figure 2).^32^ Finally, we extracted the unpermuted residuals of qT1 and qT2 values at different cortical depths within the lesion area. These were computed as follows: (*Y* − *Z*) × *g*, where *Z* is the matrix with confounds (i.e., age, sex, thickness, and curvature) and *g* is the estimated generalized linear model regression coefficients from *Y* = (*X* × *b*) + (*Z* × *g*).

This allowed computation of the *z*-score profile of each patient normed against controls, corrected for age and sex. Importantly, for this part of the analysis, we further extended the spatial sampling to 1 mm below the GM/WM border to increase sensitivity to transmantle signs.

#### 2.4.5 Testing diagnostic performance of surface qMRI features

Using Scikit-learn,^41^ a random forest classifier^42^ was trained on the gradient maps of qT1 and qT2 MRI-positive patients (*n* = 17) and controls (*n* = 31), after removing the effect of age and sex using linear regression. Random forest algorithms have important advantages in terms of ability to handle nonlinear data and robustness to noise.^43^ Default hyperparameters provided by Scikit-learn^41^ were used. Briefly, the classifier had 100 decision trees. The square root of the number of features was analyzed at each node to find the best split, and splits were evaluated using the Gini impurity criterion. The classifier employed bootstrap samples to perform resampling. qT1 and qT2 features for the whole brain were concatenated and input to the classifier for training. After training, the model’s performance was tested on qT1 and qT2 gradient maps of MRI-negative patients (*n* = 26) and controls (*n* = 14) held out from the training set.

For completeness, the same procedure was then applied to train a classifier on the data of MRI-negative patients (*n* = 26) and controls (*n* = 14). This was then tested on MRI-positive patients (*n* = 17) and controls (*n* = 31) held out from the training set.

Area under the receiver operating characteristic (ROC) curve (AUC), accuracy, sensitivity, specificity, and F1-scores were used to evaluate classification performance. A permutation-based *p*-value was calculated to assess the probability that the observed results could have been obtained by chance.

## 3 Results

### 3.1 Group differences in qT1 and qT2 at each cortical depth

#### 3.1.1 Cohort differences

**Figure 3:**
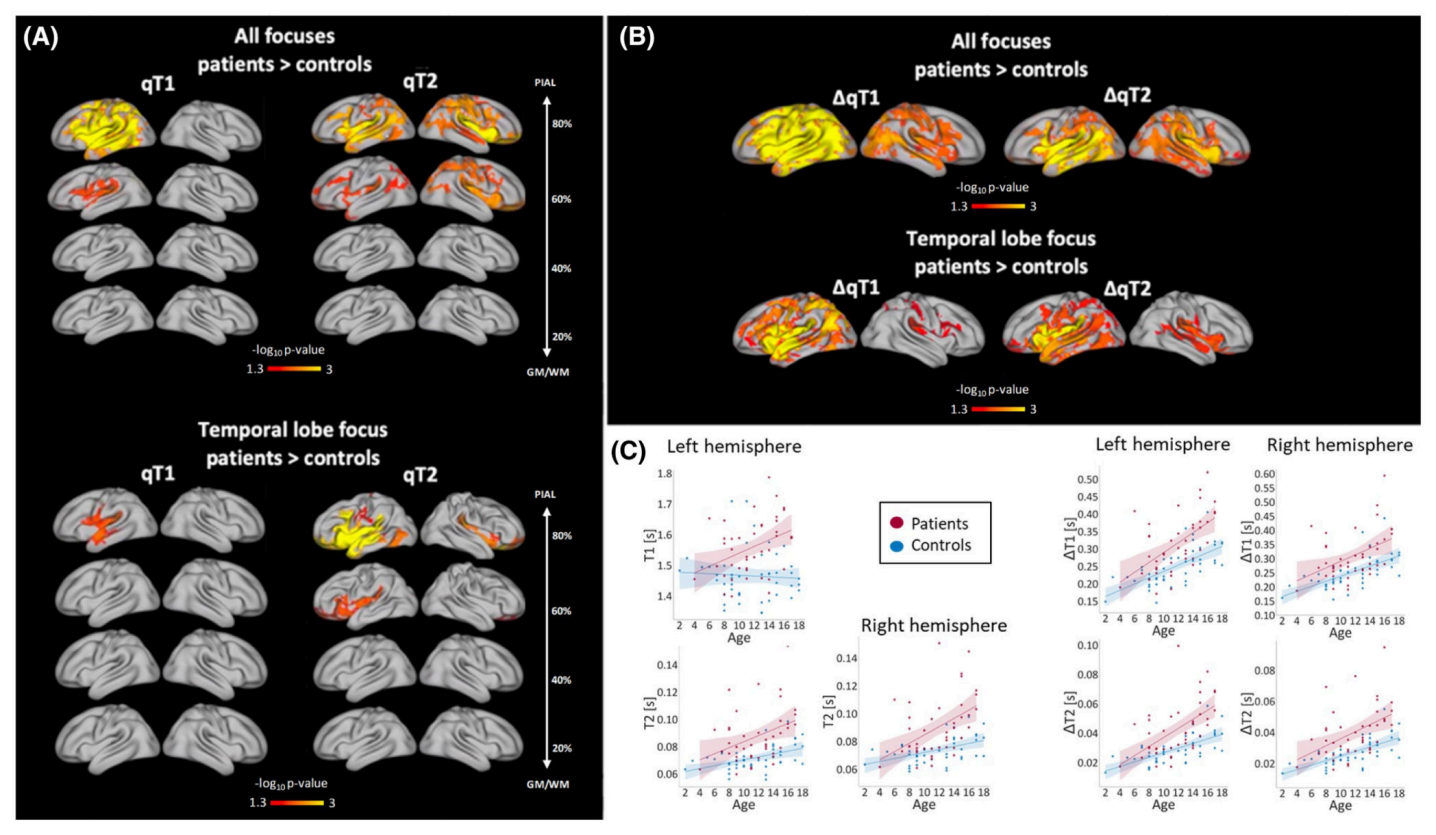
(A) Group differences between patients and healthy controls in qT1 and qT2 at each cortical depth. When comparing all patients against controls, qT1 increases were detected in the ipsilateral hemisphere at 60% and 80% depth in patients, and bilateral increases were detected in qT2 at 60% and 80% depth. When splitting patients into frontal versus temporal lobe focus, qT1 increases were detected in the ipsilateral temporal lobe in temporal lobe epilepsy (TLE) patients compared to controls. Additionally, qT2 increases were detected at 60% and 80% distance in TLE patients compared to controls. (B) Group differences in qT1 and qT2 gradients (ΔqT1 and ΔqT2). When comparing all patients against controls, widespread steeper qT1 and qT2 gradients were detected in patients across both hemispheres. When splitting patients into subgroups based on focus, widespread steeper qT1 and qT2 gradients were detected in TLE patients compared to controls. Alterations extended beyond the temporal lobe. (C) Relationship between age and average qT1 and qT2 values in patients and controls. In the left panel, average qT1 and qT2 values at 80% distance from the gray/white matter (GM/WM) border are plotted for each subject against age in areas where significant group differences were detected. On the right, average qT1 and qT2 gradient values are plotted against age for each subject, again in areas where significant group differences were detected.

Increases in qT2 at the 60% and 80% depth from the GM/WM border were detected in patients. Although effects were stronger in the ipsilateral hemisphere, increases in qT2 were bilateral. Additionally, qT1 increases were detected at 60% and 80% depth from the GM/WM border in the ipsilateral hemisphere in patients (Figure 3A, top). Trend-level differences (i.e., FWE-corrected within a contrast, but not significant after correcting across contrasts and modalities) were observed for the 40% depth for qT2 but not qT1. No differences were observed for the 20% depth. Figure 3C (left quadrant) plots subject-average qT1 and qT2 values at 80% distance in areas where significant group differences were detected, against age.

#### 3.1.2 Lobar differences

When splitting patients into frontal and temporal lobe groups, qT1 increases were detected at 80% distance from the GM/WM border in the ipsilateral temporal lobe of TLE patients versus controls. Additionally, qT2 increases were detected at 60% and 80% distance from the GM/WM border in TLE patients, especially in the ipsilateral hemisphere. Although stronger effects were detected in the temporal lobe, increases in qT2 were widespread, especially at 80% distance from the GM/WM border. Trend-level qT2 differences were observed for the 40% depth. No qT1 or qT2 differences were observed for the other depths. We detected no significant differences between frontal patients and controls, nor between the two patient subgroups (Figure 3A, bottom).

With both analytic approaches, we did not detect associations between microstructure changes and clinical variables. However, there was a positive relationship between disease duration and age in patients (rho =.59, *p* <.001), potentially indicating the presence of collinearity, which might have impacted our sensitivity to associations between microstructural changes and clinical variables.

### 3.2 Group differences in qT1 and qT2 gradients as an index of intracortical organization

Increasing qT1 and qT2 values toward the pial surface were seen across the cortex in both patients and controls.

#### 3.2.1 Cohort differences

Permutation tests revealed a significant group difference in qT1 and qT2 gradients, indicating a steeper gradient of variation across cortical depths in patients, with increasingly higher qT1 and qT2 values in the outermost cortical depths across hemispheres (Figure 3A, bottom). Effects were widespread and bilateral; however, they were stronger in the ipsilateral hemisphere. Figure 3C (right quadrant) plots subject-average qT1 and qT2 gradient values in areas where significant group differences were detected, against age.

#### 3.2.2 Lobar differences

We detected steeper qT1 and qT2 radial gradients in patients with TLE compared to controls. The changes concerned mostly the ipsilateral hemisphere and extended beyond the temporal lobe, affecting most of the cortex. We detected no significant differences between frontal patients and controls, nor between the patient subgroups, and no significant associations with clinical variables.

### 3.3 Surface-based qMRI profiles of FCD lesions

**Figure 4:**
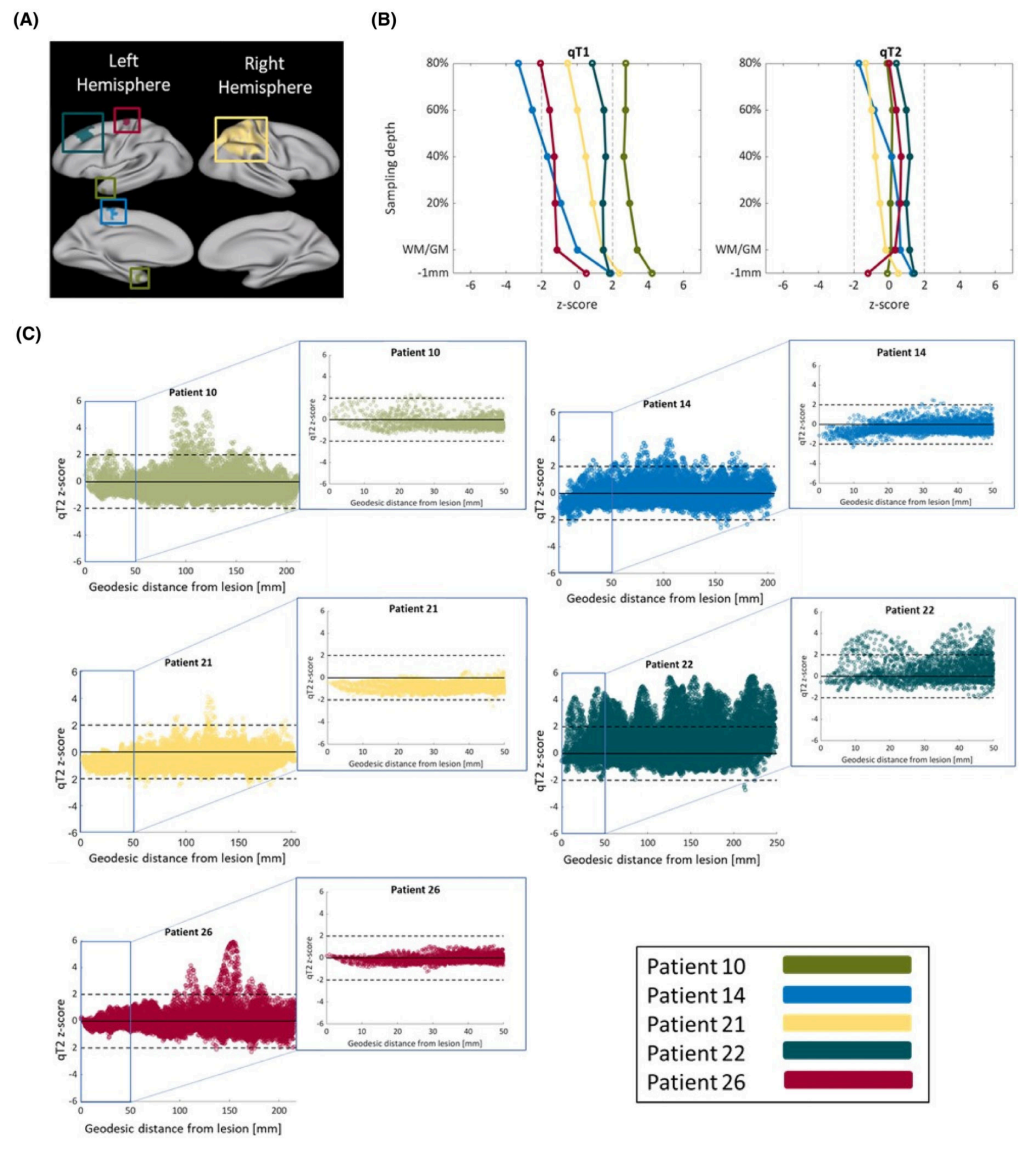
Spatial modes of qT1 and qT2 alterations are not driven by focal abnormalities. (A) Surface maps of focal cortical dysplasia (FCD) locations of all patients. Lateral and medial views are presented for both hemispheres. The FCDs were located in temporal, frontal, and parietal areas. (B) Quantitative magnetic resonance imaging *z*-score profiles of FCDs for qT1 (left) and qT2 (right). Here, each line represents the *z*-score deviation of one patient against all controls, across cortical depths. Any *z*-score greater than +2 or smaller than −2 (black dashed lines) represents a significant deviation from the control distribution. Detected changes were heterogeneous between lesions and mostly not significant. (C) qT2 *z*-score values as a function of geodesic distance from the lesions’ center at 80% cortical depth. Both cortexwise and zoomed-in views (i.e., within 50 mm of the lesion center) are plotted. In all patients, the spread of abnormalities presents an “FCD-fugal” pattern, with abnormalities affecting cortical areas remote from the FCD. WM/GM, white/gray matter.

Figure 4 illustrates the surface maps of the FCD locations, as well as the *z*-score profiles of FCDs across cortical depths, for each patient. The detected deviations from the control distribution were heterogeneous between lesions and mostly not significant, suggesting that spatial modes of qT1 and qT2 alterations are not driven by focal abnormalities.

### 3.4 Classifying epilepsy in MRI-negative patients with qMRI features

**Figure 5:**
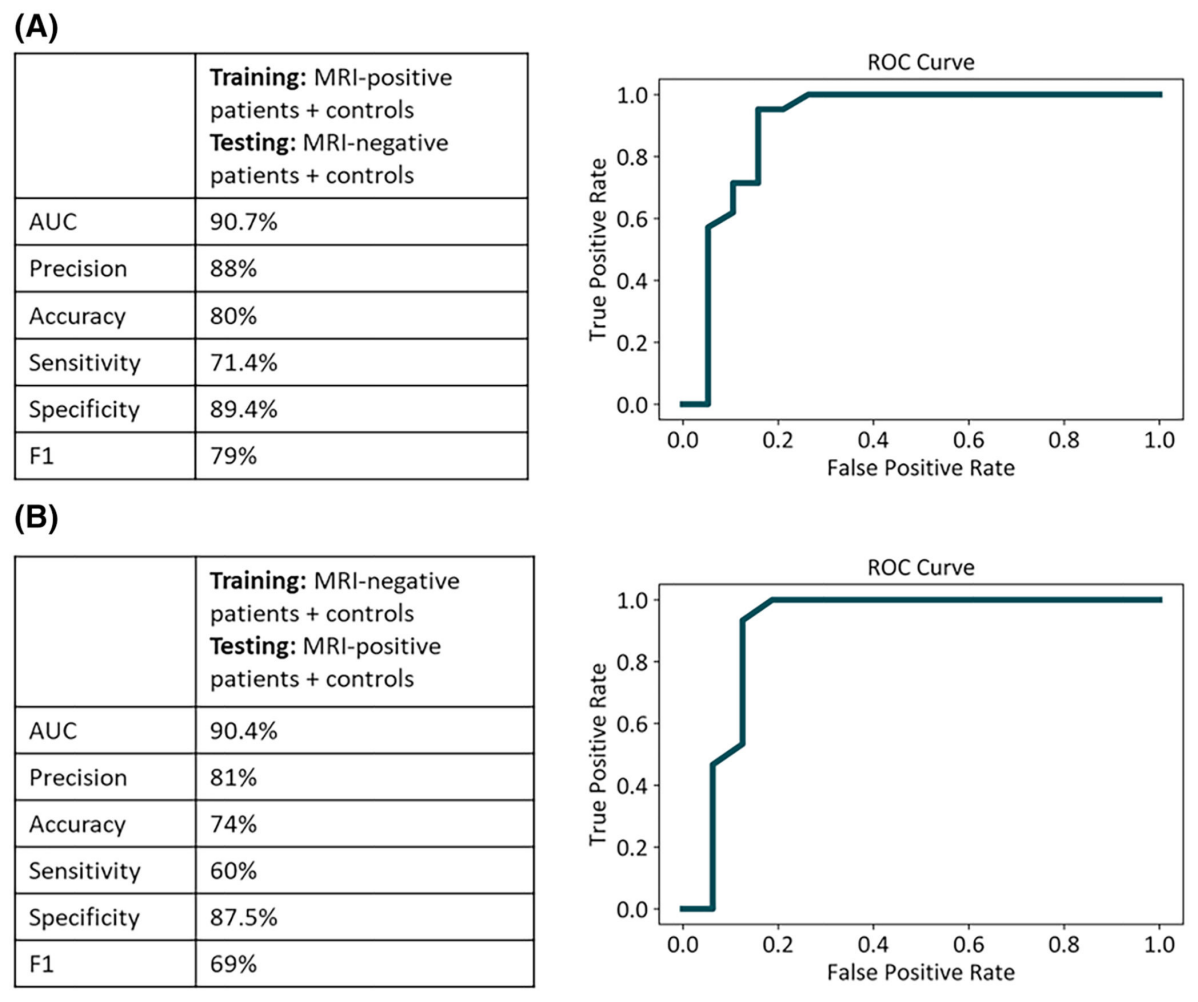
Random forest classifier performance. For both classifiers, the confusion matrix and receiver operating characteristic (ROC) curve are displayed. For both classifiers, the potential for classification could not be attributed to chance, and the *p*-value was significant. AUC, area under the curve; MRI, magnetic resonance imaging.

On a classifier trained with MRI-positive patients and controls, we had a test AUC of 90.7%, accuracy of 80%, sensitivity of 71.4% at 89.4% specificity, and an F1-score of 79% on MRI-negative patients and controls who were not part of the training group. To test robustness to training data, we flipped the training and test datasets. When the classifier was trained with MRI-negative patients and controls, we had a test AUC of 90.4%, accuracy of 74%, sensitivity of 60% at 87.5% specificity, and an F1score of 69% on MRI-positive patients and controls who were not part of the training group. The classification performance could not be attributed to chance (*p* =.001; Figure 5).

## 4 Discussion

We assessed whole brain cortical changes in children with drug-resistant focal epilepsy. The marked heterogeneity in the sample in terms of lesion/focus location and underlying pathologies could be leveraged to further understand shared patterns of abnormalities. The use of a multiparametric qMRI approach allowed moving beyond morphological markers to probe changes in cortical microstructure.^24^

Our findings indicate the presence of consistent, wide-spread, depth-mediated qT1 and qT2 increases in pediatric focal epilepsy. Additionally, they reveal significant alterations in patients’ intracortical organization,^44^ demonstrated by a radial gradient of increased qT1 and qT2 values driven from the WM/GM border. Consistent with previous evidence,^21,26,45^ cortical changes were robust to potential confounders, including age at scan and brain morphology (Figure S2), suggesting that they represent a different underlying pathophysiological mechanism. Moreover, alterations did not appear specific to, or driven by, patients’ focal cortical lesions, as demonstrated by the finding that qT1 and qT2 values within the investigated FCDs did not significantly deviate from control values in the same area. Importantly, in accord with previous studies,^12,21^ clinical parameters did not correlate with qT1 and qT2 across the cortex, implying that such microstructural alterations may be independent of ongoing disease processes.

Notably, our findings indicate that microstructural alterations in focal epilepsy are independent of seizure onset or focus laterality (Figures 3, S3). Although stronger changes were detected in the temporal lobe of TLE patients, alterations still extended to the wider cortex. Such widespread effects were particularly evident when looking at qT1 and qT2 radial gradients. Although no alterations were detected in the subgroup of patients with frontal focus, this is most likely because of the smaller size of this subgroup. Additionally, although qT1 increases at different depths predominantly affected patients’ ipsilateral hemisphere, we detected bilateral effects on the qT1 gradient over cortical depths. Finally, extensive qT2 increases were uncovered in patients across cortical depths and when looking at the radial gradient of change. Our findings are in accord with previous work demonstrating the presence of morphological alterations following a bilateral pattern and extent that is independent of the side of seizure focus,^3^ and not specific to the patients’ focal pathology.^3,4^

Importantly, we demonstrate that these widespread cortical features are sufficiently consistent across all children with epilepsy that they can act as classification features. A random forest classifier trained on whole-brain qT1 and qT2 surface maps from MRI-positive patients and controls could classify epilepsy status in MRI-negative patients and controls. Likewise, the same classifier trained on whole-brain qT1 and qT2 surface maps from MRI-negative patients and controls could classify epilepsy status in MRI-positive patients and controls. These findings suggest that there may be an imaging endophenotype of focal epilepsy, common in MRI-positive and MRI-negative cases.

There are several underlying microstructural changes that could explain the cortical qT1 and qT2 alterations observed in patients. For qT1, previous evidence suggests that this measure may be sensitive to GM myelin content.^46^ Therefore, increased qT1 values and alterations in qT1 gradients may reflect the presence of cortical myelin disturbance in pediatric focal epilepsy, mirroring pathology findings in TLE where surgical specimens had alterations in intracortical myelination and fiber arrangement, particularly in upper cortical layers.^47^

On the other hand, the qT2 depthwise and gradient increases observed in this study might be related to gliosis. Previous evidence has reported a correlation between gliosis and hippocampal subfields qT2 in TLE,^48^ although this has not yet been reported in cortical epilepsy. Gliosis on a microstructural level may reflect a remodeling process that might explain the observed qT2 increases.^21^ Alternatively, because qT2 is dependent on free water content,^24,49^ tissue damage and replacement of cells in nervous tissue by water could potentially be another mechanism leading to the increased cortical qT2 observed in patients.

From an etiological point of view, ongoing seizure activity could underly qT1 and qT2 alterations observed in this study. Accordingly, Su et al.^4^ reported limbic/paralimbic qT1 increases in children and adults with focal epilepsy that correlated with younger seizure onset age, longer epilepsy duration, and higher seizure frequency, suggesting that such changes may be related to seizure burden.

However, other previous investigations^12,21^ failed to detect a significant relationship between structural parameters and clinical seizure occurrence, and reported the lack of an association between anticonvulsive drugs and qT2 variation.^21^ Crucially, the ability of our classifier to classify young patients, and the lack of association between qT1 and qT2 changes and disease duration, age at onset, and seizure frequency, indicate that alterations may be independent of disease progression and instead might constitute antecedent microstructural alterations reflecting developmental cortical changes.^20,26^

Further supporting the hypothesis that qT1 and qT2 abnormalities may reflect precursor anomalies in brain development, a longitudinal study of children with new onset focal and idiopathic generalized epilepsy demonstrated differences in baseline GM and WM volumes of focal patients compared to controls.^50^ Furthermore, Widjaja and colleagues reported changes in cortical thickness in the right medial temporal, cingulate, and left frontal cortices in children with new onset seizures.^51^ It is possible that such early brain changes predispose patients to seizures, but this remains a challenging hypothesis to test in focal epilepsy compared to family designs possible in genetic epilepsies.^52,53^

## 5 Study Limitations And Future Directions

This study was conducted using a cross-sectional design, therefore limiting control over the confounding effect of age. Future studies assessing pediatric patients with new onset seizures longitudinally will enable better disentangling the impact of age from that of disease, as well as of recurrent seizures and long-term antiepileptic medications on the brain. Furthermore, the specificity of these global cortical changes to focal epilepsy as opposed to seizures is unclear, and inclusion of other epilepsies is needed.

Although qMRI offers several advantages over conventional qualitative imaging approaches, qT1 and qT2 are affected by several microstructural properties.^31^ Consequently, several mechanisms, such as demyelination and axonal changes, alterations in free water content, edema, and iron accumulation, may all underly the qT1 and qT2 changes observed in this study. Future studies could use susceptibility approaches to image subtle variations in cortical iron depositions and myelination, thus providing rich complementary information to the existing evidence. Additionally, the integration of qMRI with macroscale diffusion-based connectivity changes promises to better elucidate both the local- and network-level substrates of drug-resistant focal epilepsies.

Finally, the 1-mm isotropic resolution used in the present study hindered our ability to look at granular depth-dependent measures, but still allowed us to look at depth-dependent and gradient changes in the signal. Importantly, to avoid pulling data from the same voxels, and therefore assessing overlapping samples, we used the ribbon-constrained method to perform volume to surface sampling, allowing us to get weighted averages of the values sampled from a given voxel, with a different weighting for each cortical depth.

## 6 Conclusions

To conclude, we demonstrate widespread, depth-mediated qT1 and qT2 increases in the cortex of children with treatment-resistant focal epilepsy. Although the etiology of these cortical alterations is not fully understood, it is possible that such microstructural changes appear during cerebral development and represent antecedent neurobiological alterations. Accordingly, based on our findings, it is plausible to suggest that global cortical changes in surface-based qT1 and qT2 may constitute a potential imaging endophenotype of focal epilepsy, detectable with or without the presence of radiologically identified abnormalities and disease-onset.

## Supplementary Material

Supporting Information

## Figures and Tables

**Table 1:** Descriptive demographics of the study sample.

Characteristic	Healthy control participants	Patients with epilepsy	Total
*n*	46	43	89
Age at scan in years			
Mean ± SD	11.54 ± 3.96	12 ± 3.56	11.85 ± 3.76
1–5, *n*	3	1	4
6–10, *n*	18	14	32
11–15, *n*	17	20	37
16–18, *n*	8	8	16
Sex, *n*			
Male	19	21	40
Female	27	22	49
Focus laterality (hemisphere), *n*			
Left	–	25	
Right	–	18	
Focus area, ratio			
Frontal	–	13/43	
Temporal	–	22/43	
Parietal & occipital	–	1/43	
Occipital	–	3/43	
Parietal	–	4/43	
Age at onset, years			
Mean ± SD	–	6.95 ± 3.01	
Disease duration, years			
Mean ± SD	–	4.85 ± 3.16	
Seizures per year, *n*			
Mean ± SD	–	173.46 ± 288.08	

## Data Availability

The clinical and neuroimaging data used in the current paper are available from the senior author (J.O.M.) on formal request indicating name and affiliation of the researcher as well as a brief description of the intended use for the data. All requests will undergo King’s College London-regulated procedure, thus requiring submission of a Material Transfer Agreement. Full preprocessing steps and the code to run the HCP preprocessing pipeline can be found at https://github.com/Washington-University/HCPpipelines. Please also see https://github.com/Washington-University/workbench for the source code for Connectome Workbench. Other code excerpts, information regarding the analysis, or intermediary results can be made available upon request to chiara.casella@kcl.ac.uk.

## Key points

- We detect widespread quantitative T1 and T2 changes in children with focal epilepsy that vary with cortical depth.
- Cortical alterations do not associate with seizure onset, focus laterality, and ongoing disease processes.
- Cortical changes are detectable irrespective of radiologically identified abnormalities.
