# Supplementary material for: Widespread, depth-dependent cortical microstructure alterations in pediatric focal epilepsy
Source: Epilepsia. Author manuscript; Available in PMC 2024 Aug 5. (PMC7616339; doi:10.1111/epi.17861)
Supplement: Supporting Information [file EMS197250-supplement-Supporting_Information.pdf]

SUPPORTING INFORMATION

Additional supporting information can be found online in the Supporting Information section at the end of this article.

15281167, 2024, 3, Downloaded from https://onlinelibrary.wiley.com/doi/10.1111/epi.17861 by Test, Wiley Online Library on [03/07/2024]. See the Terms and Conditions (https://onlinelibrary.wiley.com/terms-and-conditions) on Wiley Online Library for rules of use; OA articles are governed by the applicable Creative Commons License
